# Journalistic Fact-Checking of Information in Pandemic: Stakeholders, Hoaxes, and Strategies to Fight Disinformation during the COVID-19 Crisis in Spain

**DOI:** 10.3390/ijerph18031227

**Published:** 2021-01-29

**Authors:** Xosé López-García, Carmen Costa-Sánchez, Ángel Vizoso

**Affiliations:** 1 Novos Medios Research Group, Universidade de Santiago de Compostela, 15782 Santiago, Spain; xose.lopez.garcia@usc.es (X.L.-G.); angel.vizoso@usc.es (Á.V.); 2 Culture and Interactive Communication Research Group, Universidade da Coruña, 15008 A Coruña, Spain

**Keywords:** COVID-19, journalism, pandemic, fact-checking, coronavirus, health, communication, Spain

## Abstract

The public health crisis created by COVID-19 represents a challenge for journalists and the media. Specialised information in healthcare and science has turned into a need to deal with the current situation as well as the demand for information by society. In this context of increased uncertainty, the circulation of fake news on social networks and messaging applications has proliferated, producing what has been known as ‘infodemic’. This paper is focused on the fact-checking of journalistic content using a combined methodology: content analysis of information denied by the main Spanish fact-checking platforms (*Maldita* and *Newtral*) and an in-depth questionnaire to these stakeholders. The results confirm the quantitative and qualitative evolution of disinformation. Quantitatively, more fact-checking is performed during the state of alarm. Qualitatively, hoaxes increase in complexity as the pandemic evolves, in such a way that disinformation engineering takes place, and it is expected to continue until the development of a vaccine.

## 1. Introduction: Journalistic Fact-Checking during the COVID-19 Crisis

Fact-checking is one of the essential elements of journalism [1,2]; it is an indispensable dimension shared by all journalism professionals throughout its modern history. Hoaxes, ever present in the history of humankind, especially during wars and between wars [3], have been a menace against true and accurate information. However, hoaxes have not been the only threat. The routine use of techniques and methods of the journalistic production to achieve objectivity as a sort of strategic ritual [4,5] was also noticed in the past century as a source of many distortions and interferences in the informative structure. Journalistic production had some weaknesses which were already being exploited in the 1990s, including the ability for the audience to do social research and use available computerized tools [6].

The critiques about how commercial demands impact the essence of media and journalism have been present in the history of technologically mediated communication [7]. The design of strategies by governments of different alignments and in different places to control the media and journalistic contents [8] is one of the aspects that have characterised the evolution of the media and journalism throughout history. These threats for journalism have been translated into the loss of information quality on different stages, something that has accentuated in the transition towards the digital scenario of the network society and that has been recognised by journalists [9]. Several empirical studies have confirmed how many journalists contribute to the education of users, while many others nurture the dormant public opinion scenarios [10].

Although objectivity is an insufficiently defined concept [11] and informative quality is still under discussion since there is no unity of criteria for its analysis, in spite of the many proposals suggested in recent years [12], both questions have actually been present in journalistic debates in the past few decades.

Digital journalism has not put an end to these debates—instead, from the very beginning, online media has shown journalistic strengths and weaknesses [13], updating and encouraging some of the old debates about the quality of many pieces of information circulating on different platforms. The quality of the news services is fairly diverse, and users do not always prefer more elaborate and solvent products [14].

Despite the influence of social networks in the core journalistic value of fact-checking [15] and the introduction of more audience-oriented editors, granting a more active role to users [16], and despite the efforts to reinvent journalism in the current societies through studies that analyse how it responds to social, cultural, political, and technological transformations and how journalism keeps true to its own principles [17], the fact is that the evolution of the network society has increased the echo of interference and disinformation. A proof of this is that the World Economic Forum considers it as one of the main threats to human society [18]. Disinformation has been ubiquitous in the communicational scenario and has positioned it in the bullseye of debates of current society, especially due to the recent pandemic [19].

From to perspective of journalism, the disinformation menace is also interpreted as a new opportunity, using up-to-date methods and techniques for creating more interdisciplinary teams that nurture the scientific dissemination processes and with the support of current technologies to reinforce procedures that guarantee the veracity of the informational pieces and contribute to their reinforcement [20].

Nonetheless, despite the elusive nature of fact-checking criteria that apply to journalistic practice and the fact that professionals often use fact-checking as a strategic ritual [21], in recent years, many of the actions to fight disinformation from the journalistic practice have been oriented to the improvement of fact-checking procedures.

Since healthy democracies need a healthy media and quality journalism [22], the search of solutions to the weaknesses of journalism in terms of verification have appeared in the form of of fact checking, which offers innovative alternatives that combine the technological and communicational dimension to deal with disinformation [23]. The purpose of fact-checkers and fact-checking organisations is to increase the knowledge through the research and dissemination of facts mentioned in statements, either published or recorded, made by political figures or any other individual whose opinions have an impact on the lives of others [24].

With the proliferation of so-called fake news [25], mainstream and digital media, as well as civic platforms, have initiated sections, work groups, or spaces targeted at the fact-checking of information published by other media, disseminated though social networks or proclaimed by the main political leaders [26].

### 1.1. Fact-Checking in Pandemic

The first studies indicate that the pandemic has led to an ‘infodemic’, defined by WHO (2020) as an overabundance of information—some accurate and some not—that makes it hard for people to find trustworthy sources and reliable guidance when they need it. Since March in Spain, an elevated circulation of fake news related to the COVID-19 virus [27] and its consequences has been detected, which is highly contagious and has an increased risk for public health [28].

In fact, in Spain, the percentage of users reporting to have seen deceiving content related to the pandemic is comparatively significant both on social networks as well as messaging apps [29].

The complicated situation created by coronavirus at all levels has generated some initial lessons on communication from the perspective of the management of the crisis and risk-related messages [30], as well as in terms of the journalistic coverage of the pandemic [31]. Furthermore, previous studies have underlined the great number of hoaxes created under a situation of uncertainty, which, in the case of those identified by the fact-checking platforms operating in Spain, are grouped into four types, ranging in a spectrum from jokes to exaggeration, decontextualization, and deception [32].

In the fight against this disinformation, the fact-checking processes have emerged and renewed, showing their usefulness during the first months of the crisis caused by COVID-19 [29], and are still necessary. Mainstream and new media, as well as new specialised platforms, have shown the relevance of their contributions, and their work can be consulted in the database generated by a collaborative process of data fact-checkers from more than seventy countries (The #CoronaVirusFacts Alliance), where all fake information identified about this pandemic can be reviewed.

### 1.2. Maldita and Newtral

*Newtral* and *Maldita* are considered the two largest journalistic fact-checking platforms in Spain due to the dimension of their work, comprising different areas, as well as due to the strong convictions of their founders about fact-checking within the Spanish democratic and civic configuration [33].

*Maldita* is defined as an independent non-profit media that does not belong to any communication group. Constituted as an association on 27 September 2018 under the name *Asociación Maldita contra la Desinformación: Periodismo, Educación, Investigación y Datos en Nuevos Formatos* (Maldita Association against Disinformation: Journalism, Education, Research, and Data in New Formats) (National Number: 616385). Today, it is transforming to become a foundation. It is integrated into the International Fact-Checking Network (IFCN), a signatory entity of its Code of Principles [34,35].

*Newtral* is a new audiovisual start-up created in 2018, founded and managed by the reporter Ana Pastor, its only shareholder. *Newtral* works in three areas: producing television programmes, working on fact-checking in the journalistic field, and denying all sorts of hoaxes and researching the protocols of artificial intelligence. It is also part of the IFCN, the purpose of which is promoting excellence and good practices in the methods and work procedures of fact-checkers, as well as to guarantee the independence of their work and the neutrality of their contents, through the fulfilment of their Code of Principles [36].

This work aims to contribute to the studies about disinformation, fake news, and data fact-checking, in a context of elevated informative interest. This document analyses the stakeholders, fake news and the fact-checking strategies developed, focusing specifically on the Spanish context and contrasting two study periods of different characteristics: the period immediately after the declaration of the state of alarm in Spain and the onset of what would be considered the “second wave”. This is the first comparative communication study made in Spain about fact-checking in the context of the COVID-19 crisis.

## 2. Materials and Methods

The analysis is based on the combination of quantitative and qualitative methods to understand the study object: fake news and the fact-checking processes conducted in Spain in two different time periods of the current pandemic caused by COVID-19.

*Newtral* and *Maldita* have been chosen as the main platforms of analysis because they are the two main agents specialised in information fact-checking, their core task [33].

Regarding methodology, there is a combination of the content analysis of the hoaxes gathered and their corresponding fact-checks and the in-depth questionnaire about the work developed in both entities during this period of the pandemic.

For the content analysis, two periods of study were considered to achieve a comparative perspective between them. The two periods of study ranged from the 15th until the 30th of March (‘acute stage’ of the healthcare crisis in Spain, because on 15th March there was the declaration of the state of alarm and the general lockdown of the population) and from the 1st to the 15th of August, which can be called the ‘chronic stage’, but where there is a rise of cases since April, therefore the media in Spain start talking about a possible second wave. Although the expression "second wave" is still used today, it was during the first fifteen days of August that the headlines of the Spanish media questioned what had been known as "rebounds" until then, and during the de-escalation phase, to call it a "second wave" instead. The Health Counsellor of the Basque Autonomous Community, one of the 17 Autonomous Communities in which Spain is organised administratively, was the first institutional spokeswoman to use the label of this new stage. In total, 146 hoaxes (N = 140) were detected, with quantitative emphasis on the first period of analysis (N_1_ = 122). The tool used in this content analysis was created ad hoc, based on previous studies [29,32,37]. This coding tool (Table A1, available at Appendix A) included two sets of variables:

1. Identification data (headline, media outlet, publication date, URL)
2. Content data, specifically:
  - Theme, open variable recategorized in a top-down process into: (a) politicians (there are political figures and their activities in the centre of disinformation); (b) measures and/or sanctions (regarding the new situation, fake information emerges about what is considered legal, illegal or expected from citizens, companies and organisations); (c) remedies and/or vaccines (protection and prevention); (d) hospitals and healthcare service (the healthcare setting turns into the focus of new disinformation); (e) incidents (alterations of the public order); (f) frauds/*phishing*; (g) education (regarding the school year, suspension or postponement of classes, etc.); (h) help and/or donation campaigns; (i) other.
  - Source/s used in the fact-checking (institutional, referential, scientific, as main categories).
  - Disinformation scope (local, regional, Spain, International, non-specifiable).
  - Typology: reconfigured (type); manufactured (type); parody. The Deepening category was added for those informative pieces oriented to the clarification of confusing information for citizens.
  - Format (image, video, infographics, text, other).
  - Motivation (variable that was also recategorized later).
  - Hoax’s expansion platform (Facebook, Twitter, YouTube, Web, WhatsApp, other, not specified).

About 10% of the sample was coded by a second researcher with a coincidence index of 97%. Cohen’s Kappa was 0.8 (almost perfect agreement).

Regarding the in-depth questionnaire (available at Appendix B), it was conducted to key figures in the contents fact-checking of both entities during pandemic: the Editorial Projects Coordinator in *Maldita* (I1) and the Editorial staff of *Newtral* (I2). This questionnaire was structured into three large sections, with a set of subthemes:

Impact of the COVID-19 crisis in the organisation and its work procedures:

1. General changes.
2. Staff changes.
3. Training and professional profiles required.
4. Impact on the work procedure.
5. Perception on performance.

Hoaxes related to the pandemic:

1. Relevant features (type, format, theme, platform…).
2. Most useful sources of information in the fact-checking processes.
3. Drivers.
4. Methodology.

Prospective:

1. Future impact in the organization.
2. Evolution of ‘infodemic’.

The subobjective of the questionnaire was to complement and to delve into the exploratory analysis made in the previous stage. The approach to the work model of this kind of recent stakeholder of the mediatic ecosystem and the possible impact generated by the pandemic is an innovative and necessary element to contextualise the fact-checking processes of information. Their self-perception as hoax experts is also interesting for the description and interpretation of disinformation pieces that emerge and circulate in the specific contextual moment, even beyond the sample analysed.

The questionnaire was self-administered and sent by e-mail and their answers were received between 15 September and 15 October 2020. A theme analysis was conducted following the recommendations of Braun and Clarke [38]. The themes detected (data driven) included: quantitative increase in the first stage (hoaxes, fact-checks, queries); changes in the fact-checking platforms (staff, processes); types of hoaxes (evolution of hoaxes); fact-checking information sources; circulation platforms; and drivers.

## 3. Results

### 3.1. Impact of COVID-19 in the Work Processes

Both platforms coincide in the existence of a growth of disinformation and the social demands about it associated to the pandemic. There are specific references to the “first wave”/”first peak of the wave” as the most outstanding temporal context.

“*The coronavirus crisis represented an exponential increase in the number of hoaxes circulating on the networks and also of our fact-checking work. Since January, and especially during the first months of the state of alarm, we have received hundreds of messages with fake information about the pandemic*”

*(I2)*

“*Increase of the workload in an exponential manner, especially during the peak of the first wave, in the months of March and April*”

*(I1)*

This has led to certain modifications in the organisation of their work processes. The staff is reorganised, reinforcements are incorporated, or the ones that already exist are intensified. Two requirements of content fact-checking companies are observed: specialisation in scientific information and the visual or graphic aspect as a dissemination element of the information. From *Maldita* (I1), it is reported that they increased and reinforced their staff (two part-time editors specialised on scientific information were hired; the working schedule of another three editors was extended and a graphic designer incorporated to the team, responsible of preparing infographics about informative pieces). In *Newtral* (I2), they opt for reorganising resources and teams, reinforcing the fact-checking section.

In addition, the increase in the social demand for contrasting information is recognised, confirmed due to two mechanisms: the increase in the queries made in the platform’s WhatsApp and the increase in the web traffic detected.

“*If we observe the traffic of users during these months, in the peak of the pandemic, Maldita’s website shifted from two million unique-users to 10 million in March and 7.5 million in May. Maldita’s registered users increased from 20,000 up to 40,000. The fact-checking demonstrated to be more necessary than ever*”

*(I1)*

“*We have witnessed an increase in the website’s traffic, becoming especially noticeable in the first months of the pandemic, after the state of alarm was declared. In the same period, the queries we received through our WhatsApp service multiplied by 16. There were days when we received about 1500 messages*”

*(I2)*

Over time, the crisis chronifies and the volume of work reduces. The fact-checking stakeholders work again on other subjects besides the pandemic. However, *Maldita* keeps part of the new staff hired: the graphic designer and one of the scientific journalists. From both platforms, it is recognised that the pandemic will continue to be the fundamental issue in the coming months.

### 3.2. Theme, Scope and Typology of the Disinformation Detected

The quantitative differences between the two periods of the study show an intensification in the number of hoaxes denied in the first stage (86.9%), just after the declaration of the state of alarm in Spain and, therefore, when the level of uncertainty among the population was higher. There are no significant differences between both platforms analysed in this sense. Professionals of said fact-checking entities recognised the “exponential” increase in their workload during the first months of the state of alarm in Spain and their reduction afterwards (see Section 3.1).

The fact-checking stakeholders recognise a thematic evolution of the hoaxes denied, differentiating three stages with diverse predominating themes, in accordance with the evolution of the situation and an escalation of the deepening of the social perception about the virus: the initial lack of knowledge, the fear of the disease, and thinking about the causes of the origin of the pandemic.

“*We have lived different disinformation phases. First, the hoaxes were based on the lack of scientific knowledge we had about the virus and the disease: false cures, false remedies... The second phase produced in Spain out of the fear about the healthcare crisis we were experiencing: fake audios about closure of hospitals or about mobile IVU at the homes of our politicians. Then, the conspiration came: the human creation of the virus or its implantation through 5G*”

*(I1)*

“*In the de-escalation stage and in this second wave, two situations coexist. The political hoaxes: those received in the mobile phone and which our politicians do not hesitate to disseminate from their positions. And the negationists: the denial of the pandemic, the anti-masks and anti-vaccine movement. They launch the message: “Do not stay at home, do not wear masks, everything is made up. The government is deceiving you”. In this case, a great danger joins polarisation and lie: the rebound*”

*(I2)*

In the second stage of the evolution of the pandemic, already in the recent context, there appear to be new versions of hoaxes that have already been treated with some kind of variation.

“*We have seen more ‘recycled’ hoaxes and variations of ones we previously denied. The hoaxes keep travelling from one country to another with small changes*”

*(I2)*

The content analysis shows the thematic predominance of the questions related to the disease, its affectation and treatment (35%), followed by the false measures and sanctions derived from the state of alarm in Spain (16.4%) or fake statements and/or political actions (12.8%), as shown in Table 1. However, there is an evolution in this main theme. In the first stage of analysis, the fake news is focused especially on false remedies and treatments, and in the second stage (August), a step forward is made and focus shifts to negative consequences for health derived from the use of preventative devices already adopted by society, such as masks or PCR tests.

**Figure 1:**
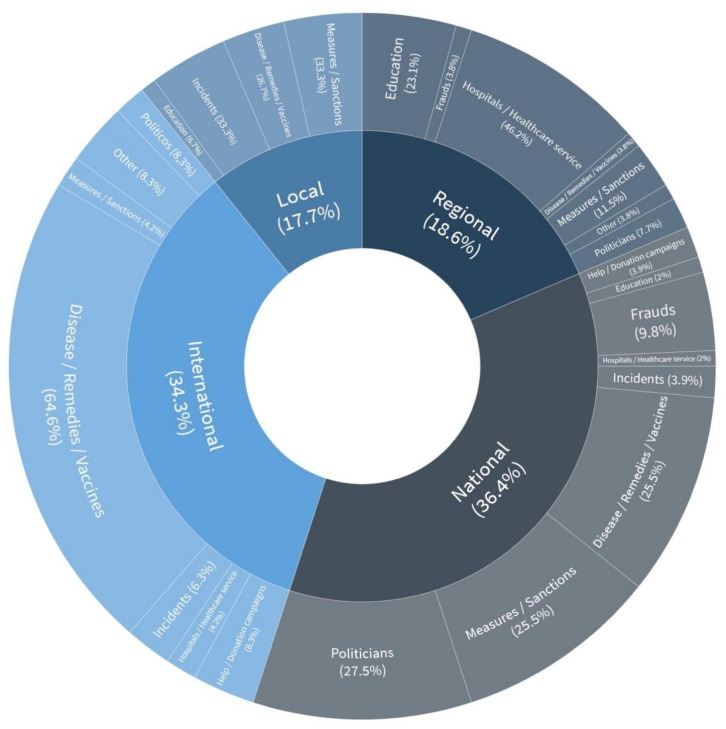
Disinformation by themes. Authors’ own elaboration.

Figure 1 shows how the field of disinformation is produced both at a national (36.2%) as well as an international scope (34.28%), leading to globalisation being seen as an element that favours the circulation of information and disinformation at all levels.

The hoaxes that have circulated the most in the international scope have been the ones related to the remedies, vaccines and the disease caused by the new virus (64.58%). China and the United States played the leading role or have been directly or indirectly linked in most disinformation. In the national scope, in Spain, there is no clear predominance, but instead hoaxes regarding politicians (27.45%), disease, vaccines and remedies (25.49%) and measures and sanctions (25.49%) have been denied. Regarding politicians, there have been fake information pieces about the main stakeholders of the political and institutional system in Spain (the president of the government and his family, vice-presidents of the government and different ministers, the monarchy and political party leaders, to a lesser extent). Regarding the sanctions and measures, the official sources are replaced to grant credibility mainly to information about fines, restrictions, and mobility schedules. In the regional scope, the predominant theme in the Autonomous Communities is hospitals and the healthcare service (46.15%). In fact, the Community of Madrid and its hospitals are mentioned in most information pieces.

Table 2 shows how, in terms of the typology of fact-checked disinformation, it is mainly manufactured content (60%), namely, created to circulate on the networks. In the reconfigured content (35%), on the other hand, there is a predominance of content for which the context has been modified to alter its meaning (24.28%). Regarding period, in the first period, the manufactured content predominates, while in the second one, the presence of manufactured content reduces and equals the reconfigured news with fake and manufactured context.

Out of all of the manufactured content, most of it (42.62%) is related to the themes of vaccines, remedies, and the disease, followed by disinformation about politicians (19.67%) and the new measures or sanctions (16.39%). Out of all of the reconfigured content, where the context has been manipulated, the predominating theme is also vaccines, remedies, and the disease (38.23%), followed in relevance by incidents (20.58%).

### 3.3. Sources of Information for Fact-Checking

An average of 2.59 sources (2.25 in *Maldita* and three in *Newtral*) are used for information fact-checking, as shown in Table 3. The themes that required a greater number of sources for fact-checking (use of sources above average) were politicians (actions and statements), in both platforms (use of three sources or more to deny them), and the vaccines, treatment and affectation of the disease in *Newtral* (3.8 sources). Regarding disinformation scope and internationally, a greater number of sources are reviewed for fact-checking.

There were essentially three types of sources used for information fact-checking: scientific and medical material (17.85%), referential and competences-based material; in relation to health (36.42%) and institutional material (73.57%), which indicates that the informative leadership lies in institutional sources. Regarding period, in the second stage of the analysis, the prevalence of scientific sources intensifies (50%), that of healthcare referential sources is maintained (44.44%), and that of institutional sources reduces (61.11%). The passage of time points to a consolidation of scientific sources in fact-checking. In addition, in August, the institutional activity reduces in Spain due to summer holidays, which may be the reason for the reduced use of institutional sources.

Nonetheless, the fact-checking stakeholders emphasise the relevance of scientists as informative sources needed for the fact-checking task, probably because they do not consider the institutional sources as something specific to the pandemic, but instead as a constant element in the usual fact-checking processes, and because the scientific sources constitute in this case the novelty regarding fact-checking in a pre-pandemic context. The relevance of the epidemiologist as a technical expert that helps to understand information is mentioned as fundamental.

“*Going to scientists and medical specialists has been essential. Epidemiologists, health officials, preventive medicine specialists, pulmonologists, virologists, internal medicine specialists, microbiologists... Without them it would have been impossible to explain contents in a simple manner for the general public and we would have taken longer for fact-checking any information (...) We signed a collaboration agreement at the beginning of the pandemic with about twenty scientific, medical societies and specialised associations so that queries were as fluent as possible, and it worked fairly well*”

*(I1)*

“*Apart from the scientific literature on the different websites of world reference, what had helped us the most was to go to specialist physicians of reference, especially epidemiologists*”

*(I2)*

The disinformation issue conditions the sources used for fact-checking. To deny fake information about the treatment and affectation of the disease, we mainly reviewed referential sources (61.22%), along with institutional (59.18%) and scientific sources (40.81%). To deny sources linked to false measures or sanctions, the institutional sources obtained almost the complete leading role (86.95%). In addition, regarding Politicians or their actions or statements (83.3%). On the platform *Maldita* (corresponding to I1), the use of scientific sources (21.51%) is superior to *Newtral* (13.11%). The percentages of the use of institutional sources are similar. The referential sources were used by *Newtral* (40.9%) to a greater extent than *Maldita* (30.91%).

### 3.4. Circulation Platforms and Format

The hoaxes circulate through diverse platforms and networks and may start on one and spread to another. The identifying actors point to WhatsApp, Twitter, and Instagram, in this order, as the main platforms where fake news circulates. The relevance of their own WhatsApp channel is outstanding, as users send them content chains that would otherwise keep hidden in the shadows.

“*The black hole of disinformation is WhatsApp, and it was so during the pandemic as well. It is the most difficult place to access and therefore the work of the Maldita Community is so important, by sending those contents circulating on WhatsApp lists to our channel so that we can proceed to fact-checking. Right after, WhatsApp, Facebook, Twitter and Instagram follow*“

*(I1)*

“*Many of the hoaxes were present on Facebook and are also disseminated through Twitter and Instagram, but also through websites. A significant proportion of hoaxes detected come through our fact-checking service in WhatsApp, where users send us chains shared on this platform*”

*(I2)*

The data indicate WhatsApp (34%), Twitter (20.71%), Facebook (13.57%), the Web (5%), Instagram (1.42%) or YouTube (1.42%). About 32.62% of fact-checks are not specified or a general expression is used that does not allow us to code a specific social network or platform.

Regarding the format, text was the predominating fake news format (45.71%). About 29.78% of disinformation was presented in an image format. Video format comprised about 14.28% of the information. Other formats, such as audio (3.57%), were circulated less often.

### 3.5. Drivers

The drivers behind disinformation are not easy to identify in all cases (13.57%). The analysis indicates that the main motivation is to create confusion and generate disorder, promoting confusion or indignation or inadequate behaviour in society (55.71Political drivers are the second most common (22.85%). Journalistic errors (4.28%) are more abundant than the economic drivers (3.57%). Journalistic errors are specially linked to the theme of measures and/or sanctions, which have been approved or not with the published features.

The fact-checking stakeholders highlight evil, political, and economic interests as the main drivers behind hoaxes.

“*There are people who share fake contents due to political reasons, to grab greater attention toward their social network profiles, others do so in order to get clicks on their websites and increase the monetisation of the ads included therein...*”

*(I1)*

“*We have identified three main drivers, common to all hoaxes. First, the economic driver, related to the number of clicks. An example of this are the fake headlines that do not have any relation to the information contents. Second, the ideological driver, also related to political motivations and with different campaigns initiated. And last, evil, mere evil. The human being is the only animal with the notion of doing evil and it is a scourge that also impacts disinformation*”

*(I2)*

The political drivers appear to be noticeably linked to the Spanish context (59.37%). The general drivers of disorder, confusion and indignation appear in the international scenario (42.3%). Regarding themes, the motivation of disorder and confusion appears in association with the following themes: treatment, disease, and vaccines (37.17%), measures and sanctions (14.1%), and incidents (12.82%).

### 3.6. Prospective

The fact-checking stakeholders point to the maintenance of the focus of their activity during pandemic, at least until there is a vaccine against the virus.

In addition, it is outstanding that the senders of disinformation are organising and there is the danger of negationists in the expansion of fake content in the society and beyond the national frontiers.

“*The pandemic will keep being the informative content that concerns everyone the most. Unfortunately, the hoaxes of anti-vaccines individuals are contents we still need to deny*”

*(I1)*

“*We are worried about the anti-scientific and negationist way of thinking observed in Spain, the public convocations and their capacity to mobilise people. It is not an issue of three isolated haters on Twitter or Facebook. They feel supported by public figures that disseminate those conspiration theories without any scientific ground whatsoever and see their reflection in other countries, where they are also organising*”

*(I2)*

Regarding the organisational level, the continuity of the teleworking system is outstanding. A system implemented during the first state of alarm, as well as the use of artificial intelligence tools, helped to organise the queries received given the case of a new increase in demand.

“*About how we would evolve as an organisation, in Maldita.es we will keep teleworking while the accumulated incidence is still that high*”

*(I1)*

“*Now, we have more artificial intelligence tools that the Engineers team of Newtral has prepared in order to easily escalate the volume of messages we receive given the case there is another wave like the one experienced back in March*”

*(I2)*

## 4. Discussion and Conclusions

This study confirms that, in the context of uncertainty, the disinformation phenomenon multiplies and expands.

The content fact-checking platforms have been supported in their activity by the social demand for delving into and fact-checking contradictory, confusing, or directly false messages that have increased since the declaration of the pandemic. The role of new stakeholders in the Spanish media ecosystem is confirmed, positioning them side by side with the media, based on the social listening and the citizens’ agenda. This indirectly represents a critique and a challenge to informative media, because the information market has new stakeholders that analyse and contrast the present from the perspective of citizens’ interest, rather than sources of journalism [39]. In the culture of convergence [40], the coexistence between mainstream and new platforms remains, considering the specificity of the activity in the latter (they serve complementary purposes—their use is not a substitute), but the new field of information has gained a leading role and acceptance, and has strengthened due to the pandemic.

Furthermore, the analysis indicates there has been a quantitative and qualitative evolution of themes over time. In fact, this is the first study that analyses the quantitative and quantitative evolution, over time, of disinformation about COVID-19 and all its consequences.

Quantitatively speaking, the volume of hoaxes is quite superior in the first stage. Then, it stabilises, in a parallel evolution to the evolution of the socio-healthcare status in Spain, namely, after the de-escalation (end of lockdown, opening frontiers, the operation resuming of the services sector). Following these arguments, a third period of analysis in the current situation (declaration of a new state of alarm since 25 October in Spain) points to a new increase in disinformation, in an oscillating evolution depending on the situation and the political, socio-healthcare, and economic measures adopted—namely, the amount of fake news and the worsening or improvement of the situation correlate positively. Likewise, this research line must continue and compare with other media, cultural, and socio-healthcare contexts.

Qualitatively speaking, hoaxes also evolve. The predominating theme is still what generates more concern among citizens: the disease, how to prevent it and finding a cure. Therefore, there is emphasis on the existence of a vaccine as the main event that could stop the expansion of disinformation. If there is a vaccine, fear and the social concern reduce. In these themes, disinformation becomes complex, and the focus is placed on tests and mechanisms that have already been socially incorporated, such as masks and diagnosis tests. The concept of disinformation engineering is suggested to refer to the evolution of hoaxes in the first stage into more complex disinformation pieces, adapted to the context and re-elaborated by organised authors to increase the credibility outlook, from simpler elements and stakeholders to the re-elaboration of themes from apparently more solid stakeholders.

“The hoaxes disseminated by specific group have gained strength, like the collective who have named themselves as ‘Médicos por la verdad’ [Physicians for truth]”. The first hoaxes we received from some physicians belonging to that group were of individual authorship. Now, they have grouped and send more elaborated hoaxes, with much more scientific terminology and even use their own heading

*(I2)*

Moreover, globalisation creates a unique scenario, where common concerns about the virus and its consequences represent a favourable context for disinformation that works in different cultural contexts. As mentioned in previous studies, in the initial context of the COVID-19 pandemic, the research into treatments and preventive measures against coronavirus is managed worldwide and the interest in these information pieces becomes global [32]. In the Spanish cultural context, the themes diversify, and political drivers come to play, namely, disinformation serves as an opposition that circulates through informal channels, contrary to the institutional management of the pandemic. The interests behind the authorship of disinformation are not easy to identify. The generation of a transnational situation of confusion, fear, or concern could respond to drivers that, initially, and after an isolated analysis of each hoax, might not emerge clearly, which does not mean there might be a possible benefit behind it.

The platforms that work as broadcasting bridges for hoaxes are mainly closed social networks, such as WhatsApp. This leads to a greater discretion in the circulation of disinformation and to a greater level of difficulty when it comes to screening and management. The fact-checking platforms have successfully implemented their WhatsApp channels to gather queries and breach social disinformation chains through their informative pieces [41]. Previous studies indicated that citizens share content out of concern for their kin [19]. Posting the information through the same channel it has expanded from represents a counteraction of the possible effects caused. Quantitatively, Twitter is the other most outstanding platform. In Spain, the use of Twitter is linked to users interested in current events with a certain preference for political and social questions in an agenda of themes clearly determined by the media [42].

The scientific specialisation of fact-checking stakeholders and the consultation to the medical and scientific specialists have been key elements in the information fact-checking processes developed. Nonetheless, the institutional source keeps playing an outstanding role in the decisions generated at the governmental level and in the association of the healthcare themes with other fields of management in the situation created. On the other hand, the temporariness of the scientific studies about the virus also led to a necessary combination of technical sources, institutions of reference (in the area of health), and institutional sources in other levels of management.

Likewise, this work entails some limitations. Research limitations are those linked to the establishment of the time period (limited to one month of fieldwork), therefore the continuation of the analysis in following stages in the evolution of the pandemic could contribute to understanding how disinformation evolves. The comparison versus other cultural and media contexts would be interesting as well. Thus, the continuation of the present line of research towards future analyses is of interest. Furthermore, the Centro de Investigaciones Sociológicas—CIS (Sociological Research Centre) [43] has detected that more than one third of citizens in Spain have an attitude of increasing mistrust towards the virus vaccine. The incidence of fake news in this social perception must be investigated. Months are still needed to achieve a vaccine that generates immunity. Disinformation engineering negatively contributes to the atmosphere of reliability needed for the management of a crisis of these characteristics [44].

## Figures and Tables

**Table 1:** Evolution of the hoax themes in the two stages of analysis.

Themes	Onset of First Wave (%)	Onset of Second Wave (%)
Politicians	12.80	5.55
Measures and sanctions	16.41	16.60
Disease, remedies, and vaccines	35.00	44.50
Hospitals and healthcare service	10.64	11.11
Incidents	4.91	22.20
Frauds	4.91	0
Education	6.55	0
Help and donation campaigns	6.91	0
Other	4.00	0
Total	100	100

Authors’ own elaboration.

**Table 2:** Typologies of hoaxes in the two stages of analysis.

Types	Onset First Wave (*%*)	Onset Second Wave (*%*)
**Reconfigured**	**32.80**	**50.0**
Content missing	10.00	0
Fake content	62.50	100
Manipulated content	27.50	0
**Manufactured**	**61.50**	**50**
Manufactured	70.00	88.90
Fictitious	29.30	11.10
**Parody**	**0**	**0**
**Deepening**	**5.70**	**0**

Authors’ own elaboration.

**Table 3:** Number of sources used to fact-checking of each theme.

Themes	S(N)	S(M)
Politicians	4.25	3
Measures and sanctions	2.50	1.75
Disease, remedies, and vaccines	3.88	2.51
Hospitals and healthcare service	2.50	1.43
Incidents	1.50	1.85
Frauds	2	1.75
Education	2.20	1.3
Help and donation campaigns	2	1

S(N) = average of sources/fact-checks in *Newtral;* S(M) = average of sources/fact-checking in *Maldita*. Authors’ own elaboration.

## Data Availability

The data presented in this study are available on request from the corresponding author. The data are not publicly available due to it is part of an ongoing research.

## Appendix A

**Table A1:** Analysis card.

Title	
**Date**	
**Media outlet**	Maldita
	Newtral
**URL**	
**Theme**	Politicians
	Measures and sanctions
	Disease, remedies, and vaccines
	Hospitals and healthcare service
	Incidents
	Frauds/Phishing
	Education
	Help and donation campaigns
	Other
**Number of sources**	
**Source type**	Institutional
	Referential
	Scientific
	
**Typology of misinformation**	Reconfigured
	Manufactured
	Parody
	Deepening
	
**Format**	Image
	Video
	Infographics
	Text
	Other
	
**Hoax’s expansion platform**	Facebook
	Twitter
	YouTube
	Web
	WhatsApp
	Other
	Not Specified

Authors’ own elaboration.

## Appendix B

Questionnaire about the coverage of disinformation during the COVID-19 pandemic at *Newtral* and *Maldita*.

### Appendix B.1. Impact of the COVID-19 Crisis on the Media Outlet

How has the coronavirus crisis affected the work at *Newtral*/*Maldita*?

Have you detected a greater workload than in previous stages?

Has it been necessary to hire more staff or incorporate external collaborators?

How many people are working on average to verify information?

Has this number been kept for misinformation on COVID-19?

Has any staff been needed with a different type of specialization or profile than those already in the newsroom?

If so, what type of training and skills were required?

If so, have these professionals been kept on staff?

Has any training or retraining been necessary to deal with the research on the specific topic of COVID-19?

To what extent has journalistic research on the coronavirus affected the usual work process in other areas?

Has the passage of time from March to the present time (September 2020) meant any change or evolution in the work with information about the coronavirus?

What has been the most complex aspect of debunking misinformation about COVID-19?

Is there a before and after in *Newtral*/*Maldita* with the spread of the pandemic in Spain? If so, in what sense?

### Appendix B.2. Hoaxes about COVID-19

What kind of hoaxes have been the predominant ones?

On what subject (or subjects) has the most misinformation been generated?

Which sources have been most useful for their verification?

On what platforms have you located the highest number of hoaxes linked to the pandemic?

How do you select the content that is going to be debunked?

What channels do you consider most effective for the dissemination of the fact checks carried out?

Is there greater public interest (visits to the website, reactions on social media, and so on) in the verification of information linked to this issue than in others?

What motivation have you detected behind most of the hoaxes located? Political, economic, certain profile of actor.

How do you think your work will evolve in terms of news coverage of this issue in the coming months?
